# Musculoskeletal Disorders: Prevalence and Associated Factors among District Hospital Nurses in Haiphong, Vietnam

**DOI:** 10.1155/2018/3162564

**Published:** 2018-08-26

**Authors:** Hoang Duc Luan, Nguyen Thanh Hai, Pham Thu Xanh, Hoang Thi Giang, Pham Van Thuc, Nguyen Mai Hong, Pham Minh Khue

**Affiliations:** ^1^Phu Tho College of Medicine and Pharmacy, Vietnam; ^2^Faculty of Public Health, Haiphong University of Medicine and Pharmacy, Vietnam; ^3^Haiphong Department of Health, Haiphong, Vietnam; ^4^Rheumatology Department, Bach Mai Hospital, Vietnam

## Abstract

**Background:**

Musculoskeletal disorders (MSDs) are a major occupational health problematic among healthcare workers, and the prevalence is especially high among nurses. In high income countries, the prevention of MSDs is an occupational health priority. But in Vietnam, there is no data available among health professionals.

**Objectives:**

To determine the prevalence and associated factors of musculoskeletal disorders among district hospital nurses in Haiphong city.

**Material and Methods:**

A cross-sectional study was conducted on 1179 nurses working in 15 district hospitals using the Standardized Nordic Questionnaire.

**Results:**

A very high prevalence of MSDs in the past 12 months (74.7%) and during the last 7 days (41.1%), with the two most common sites being the low back (44.4%) and neck (44.1%), was found; 37.8% complained that MSDs symptoms limit their work. When analyzing factors related to MSDs, the results showed that women were 2.1 times more likely to develop MSDs than men; people with a previous history of MSDs were more likely to develop MSDs symptoms in the past 12 months than those with no history (OR = 7.1); nurses with symptoms of psychological distress and frequent absenteeism in the workplace had a higher prevalence of MSDs compared to the rest (*p*<0.001).

**Conclusions:**

Due to the high prevalence of MSDs among nurses in district hospitals in Haiphong, preventive actions are needed to improve the working conditions and to raise the awareness of nurses about MSDs prevention.

## 1. Introduction

Musculoskeletal disorders (MSDs) are a widespread and increasing occupational health problems in the workplace worldwide. The causes of work-related MSDs are usually multifactorial including physical, ergonomic, and psychosocial factor [1]. MSDs usually occur in workers who have excessive repetition, awkward postures, and heavy lifting [2]. The International Labour Organization (ILO) and the World Health Organization (WHO) regard MSDs as a work-related disease, which is also referred to as a “new epidemic” that should be researched and solved. MSDs have a huge impact on work-related absence and a high proportion of days lost is due to MSDs [1]. Therefore, it not only affects the health of workers but also creates a burden on the health system, on the businesses economic, and on the social costs to deal with their consequences [1, 3]. MSDs prophylaxis is needed in many countries to allow workers to avoid the symptoms of MSDs, improve working productivity, and reduce the burden on medical systems at the same time [4, 5]. In developed countries, many programs for the prevention of MSDs have been applied on workplace [6].

In the healthcare sector, occupational musculoskeletal disorders are common, with prevalence rates of work-related MSDs reported from 28% to 96% over a one-year time period [7], including those among nurses. According to reported data all over the world, nurses have a very high prevalence of MSDs, for example, in Europe, from 10% to 50% in France [8], 89% in Portugal [9], and 85% in Macedonia [10]; in the Americas, from 35.1% to 47% in USA [11] and from 32.8% to 57.1% in Brazil [12]; in Africa, 80.8% in Uganda [13]; and in our Asia, 78.6% in China [14], 85% in Saudi Arabia [15], and 88% in Iran [16].

In Vietnam, although the occupational health sector is still underdeveloped, occupational diseases and their prevention are increasingly concerned. Currently, the list of occupational diseases covered by insurance has expanded to 34 [17]. However, MSDs are not included in this list. Many occupational disease prevention programs have been implemented in different work environments, including the medical milieu. Contrariwise, there was only one recent and unique study ever about MSDs among workers in the health sector in Vietnam in 2015 that showed a prevalence of MSDs over the past twelve months among nurses at Viettiep hospital, the largest provincial hospital in Haiphong in the northern coastal region of Vietnam, which was very high (81%) [18], and many related factors may have affected these disorders [19]. This suggests that the problem of MSDs among nurses in Vietnam can be very large. However, in order to have a comprehensive picture of MSDs among nurses, this study is to assess the current status and risk factors affecting MSDs among nurses at the district hospitals of Haiphong.

## 2. Materials and Methods

### 2.1. Study Design

A cross-sectional study was designed to determine the prevalence of musculoskeletal disorders (MSDs) and their related factors among nurses working in district hospitals of Haiphong, Vietnam, from January to June 2017.

### 2.2. Sample Size and Recruitment of Study Subjects

Study subjects included all of nurses working in all of 15 hospitals in districts of Haiphong city, with the following requirements: nursing degree, working time of 12 months or more (this period to ensure an adaptation to the working environment), and agree to participate in this study. Exclusion criteria were as follows: refusal of participation in this study, absence at work at the interview time, or a seniority of less than 12 months. A total of 1179/1279 (participation rate was 92.2%) nurses were surveyed.

### 2.3. Research Instrument and Data Collection

Data were collected through four questionnaires.

(1) A sociodemographic questionnaire was used to collect some general information of participants such as age, gender, height, weight, marital status, average income, and history of musculoskeletal diseases. We also collected information on their working characteristics: working location, department, seniority, working intensity, duration of shift work, and so forth.

(2) Standardized Nordic Questionnaire was developed by Kuorinka et al. in 1987 and was divided into two main parts. The first part evaluates general health problems of musculoskeletal system at different positions on the body during the last 12 months and within the last 7 days. The second part assesses the specific problem of MSDs in each position as well as the consequences it brings to work and the life of the respondent [20]. This questionnaire has been used extensively in many countries around the world for the MSDs study in various groups of subjects and among nursing in special [21].

(3) Psychological distress of Kessler questionnaire (K6) was used to evaluate psychological factors influencing the occurrence of MSDs. This questionnaire has been translated and validated into Vietnamese by Nguyen Thanh Hai et al. in 2013 [22]. The short questionnaire included six questions about a person's emotional state, proven to be able to categorize distress levels for respondents and recommended for use by WHO [23].

(4) The questionnaire evaluating absenteeism at work was developed by the French National Agency for the Improvement of Working Conditions [24], which was used to assess the absence in the past 12 months and allows evaluation of the impact of MSDs on the nurse's work. This survey form consists of 5 questions informing on the number of days off, total number of days off, reasons of absenteeism, and desire to change career or job placement. The Vietnamese version of this questionnaire has been used in several studies in Vietnam [25].

These questionnaires were used by our researcher for direct interviews with the nurses participating in the study. Each interview ranged from 30 to 45 minutes.

### 2.4. Statistical Analysis

The data was imported and analyzed using SPSS 19.0 version software. We used algorithms for descriptive statistics (percentage, mean, median, standard deviation, and so forth) to describe the sociological characteristics of the participants. The prevalence of MSDs was calculated as the percentage of nurses who developed symptoms of MSDs in at least one of the nine positions on the body (showed in the Nordic Questionnaire). We used chi-squared test when comparing two percentages and* t*-test when comparing two means. Multiple logistic regressions were used to analyze and identify factors associated with MSDs. The models were checked for fitness using Hosmer-Lemeshow's goodness-of-fit test. All analyses were carried out in the Faculty of Public Health, Haiphong University of Medicine and Pharmacy. The level of significance was set at a p-value of less than 0.05.

### 2.5. Ethics

The study was approved by the Hai Phong University of Medicine and Pharmacy Institutional Review Board and authorized by the Hai Phong Department of Health to implement at its district hospitals. All nurses participated in the survey were informed and signed the consent of participating in the survey.

## 3. Results

The study involved a total of 1179 nurses working in 15 district hospitals in Hai Phong city, with a participation rate of 92.2%. The number of participants in Thuy Nguyen hospital is highest with 230 nurses, while this number in Cat Hai general district hospital is lowest (16 nurses). The average age of nurses was 32.6 years (in ranging from 19 to 60, SD ± 7.7 years). More than 4/5 of the nurses were female (958 females versus 221 males). Most of them have BMI in normal range (71.4%), underweight and overweight were 11.4% and 12.0%, respectively, and the rest was obesity (5.2%). More than half of them had a monthly income from 3 million to 5 million Vietnam dongs. A significant number of nurses were married (82.4%), and the percentage of participants had at least one child was exactly 79.1%. The average of members in a family was 4.2 persons. A large number of them (90,1%) were right-handed people. Furthermore, the people who have experienced musculoskeletal diseases were 11.2% (Table 1).

In terms of working position, nurses were divided into two types of jobs: administrative (9.4%) and clinical (90.6%). Nurses who frequently work at least 10 hours each day was 37.2%. The number of nurses who have been taking responsibilities on their duty was 68.3% with the distance between two duties being 5.2 days (SD ± 1.5 days). The average time spent on the work per week was around 49.5 hours (SD ± 11.3 hours). The average of anxiety score was 4.5 (SD ± 3.7) (Table 2).

Table 3 shows the data about the percentage nurses who were absent in working place during the last 12 months; it was 48.6%. The average of absence day were 24.7 days (SD ± 52.8 days), while their median was only 5 days. In detail, the reasons for their absence were commonly involved in their own families (family work, maternity leave, and so forth) which accounted for 90.1% of nurses, followed by short-term illness (8.6%). A few of them (6.5%) considered that the working conditions were a major reason for their absence and there was only a very little proportion of people who wanted to be sent to another position or change their jobs.

**Figure 1:**
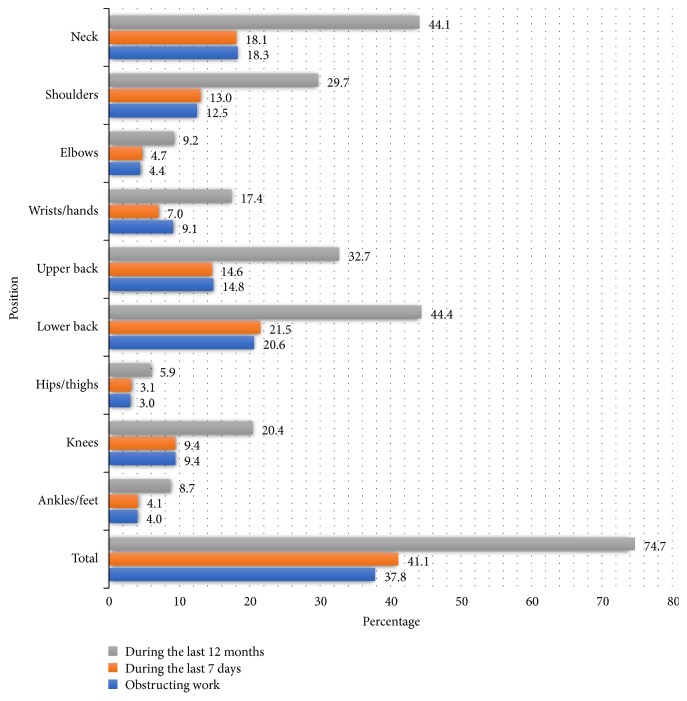
Types of musculoskeletal disorders among nurses in Haiphong, 2017.

According to the results from Nordic Questionnaire, the study revealed that the percentage of nurses suffered from MSDs during the last 12 months was 74.7%. In addition, the symptoms in the low back and the neck were highest at 44.4% and 44.1%, respectively. Around 37.8% of nurses reported that MSDs obstructed their works. 41.1% of nurses had MSDs symptoms during the last 7 days (Figure 1).

**Figure 2:**
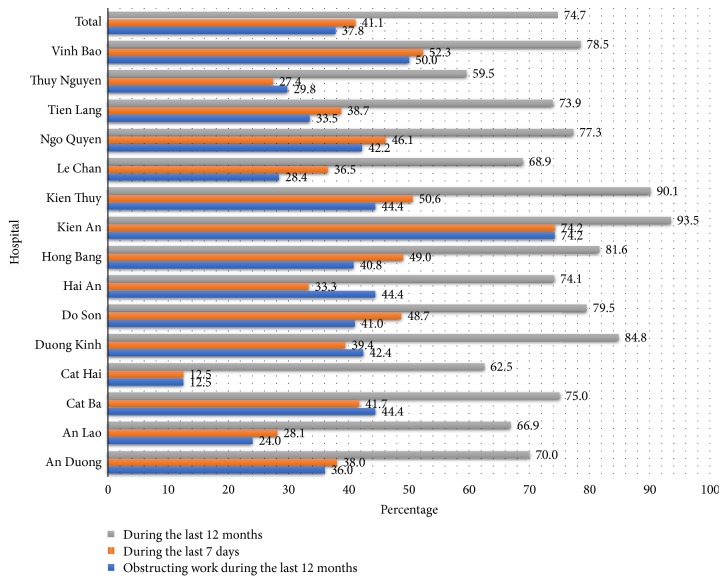
Musculoskeletal disorders characteristics among nurses by hospitals, Haiphong, 2017.

In Figure 2, sorted by hospital, Kien An and Kien Thuy were two hospitals in which nurses had the highest prevalence of MSDs during the last year, 93.5% and 90.1%, respectively. Meanwhile, this proportion of nurses working at Thuy Nguyen hospital was the lowest (59.9%).

In order to find out some related factors of MSDs, multivariate statistics were used (Table 4). The results indicated that age, history of musculoskeletal disease, anxiety, and absenteeism in the workplace were statistically significant factors in MSDs during the last 12 months among nursing. In detail, the odds of MSDs in women are 1.1 times greater than in men (*p*<0.001); participants who had a medical history related to musculoskeletal disease previously were easier to develop MSDs than those who did not (OR = 7.1;* p*<0.001); the nurses with anxiety in their lives and who have been absent at work will have higher prevalence of MSDs than those who did not have these elements (all of* p-*value were less than 0.001).

## 4. Discussion

Our study included a total of 1179 nurses currently working in 15 district hospitals in Hai Phong City, Vietnam. Work-related MSDs on the past 12 months were observed in 881 nurses (74.7%) and during the last 7 days were 41.1% and these disorders obstruct the work of 37.8% of nurses. The most common site affected was the lower back in 44.4% and the neck in 44.1%, followed by the upper back (32.7%) and the shoulder (29.7%). The significant related factors were sex (female), having a history of musculoskeletal disease, anxiety, and absenteeism.

Numerous previously studies throughout the world have shown the very different prevalence of MSDs on nurses over a 12-month period. This result was relatively similar to the other studies on nursing such as 79.5% in Turkey [26], 76% in India [27], 76.2% in long-term study from 2004 to 2010 in 3915 nurses in Taiwan [28], 70% in Poland [29], 78% in Nigeria [30], and 79.5% in China [14]. However, this result was lower than those observed in Uganda in 2013 among 755 nurses (80,8%) [13], in Estonia (84%) [31], 89% in Portugal [9], in Macedonia (85%) [10], and 80.8% in Uganda [13] and, in our Asia, there were Saudi Arabia (85%) [15], Iran (88%) [16], and Japan (85.5%) [32]. On the contrary, this prevalence was higher than that in France (from 10% to 50%) [8], in USA (from 35.1% to 47%) [11], and (from 32.8% to 57.1%) in Brazil [12]; in Pakistan (31.6%) [33], in Thailand (47.8%) [34], and in Malaysia (from 35.3% to 48.9%) [35]. There were possibly two reasons for these differences: firstly, it depends on the nursing work condition and characteristics of each continent, each country, and each region; secondary, a higher frequency is contributed by the number of symptoms of MSDs which appears in the assessment questionnaire. In this study, we used the Standardized Nordic Questionnaire with 3 references symptoms: ache, pain, and discomfort, while others symptoms may be included: numbness, stiffness, and so forth.

The most common site affected in this study was the lower back (44.4%) and the neck (44.1%). The results of some studies in Asia are comparable to this result; for example, in Pakistan in 2015, it was illustrated that around 49.7% of nurses faced MSDs in their lumbar, and 35.4% of them complained about MSDs in their shoulders [36]; another study in Iran and in Hong Kong saw the same picture with 40% and 42%, respectively, of nurses reporting MSDs in their lumbar [37, 38]; and one study in Nigeria (in Africa) showed that the rate of MSDs in lower back was 44.1% [30]. Although most studies have shown that lower back was the most common site, this prevalence was still modest when compared to that from other studies in Asia: in Japan (lower back 71.3%) [32], in Iran (73.2% in 2010 and 65.3% in 2014) [16, 39], in China (64.83%) [14], and in Saudi Arabia (65.7%) [15]; and this was similar to other studies in Europe: in Portugal (60.9% in 2015 and 63.1% in 2017) [9, 40] and in Slovenia (85.9%) [41]. Neck was also one of the most common sites of MSDs. Results in this study are similar to those of some other studies such as 46.3% in Iran [39], 42.8% in China [42], and 48.94% in Malaysia [35].

When studying the factors associated with MSDs among nurses, the results indicated several related factors that were statistically significant. Regarding gender, the odds of disorder in female are 1.1 times greater than male. This can be understood as women have lower adaptive status than men in patient-related activities, such as patient transport and infusion. History of musculoskeletal diseases in the past has been a factor in the development of MSDs. When an individual suffers from previous musculoskeletal diseases, the adaptive capacity of the musculoskeletal system to activity will be reduced; combined with inappropriate manipulations and postures in working, these conditions will motivate and generate the manifestations of MSDs. The absence at the workplaces was also a related factor to MSDs. Our results shown that this absence was one of potential risks for occupational MSDs. But within the scope of this article, it was also a limitation of this study that we have not considered the opposite, meaning the impact of MSDs on workplace absences and other consequences. In the literature, European Agency for Safety and Health at Work has demonstrated in their report in 2010 that “a high proportion of days lost in the Member States of the European Union is due to MSDs and absences are often long” [1]. In order to establish this conclusion, we need to conduct further study in the future. The last related factor that we have identified in the analytical model was anxiety. It was evident that the level of anxiety in daily life was one of the risk factors of MSDs. When a nurse has anxiety issues in common life, for example, about her/his family and her/his difficulties in work as well as in life, these will follow them to the work environment and affect the ability and concentration in work and the operation will no longer accurate because they lose their attention and cannot focus on their work. It can be a potential reason for MSDs. Anxiety was known to be associated with musculoskeletal disorders among healthcare workers including nurses [34, 43, 44]. From a psychological perspective, looking after patients with chronic diseases is associated with anxiety [45]. Biologically, anxiety is associated with inflammation [46, 47]. Nevertheless, majority of people suffering from anxiety fail to acknowledge the underlying psychiatric conditions and result in delayed treatment. It is important to offer early intervention to treat anxiety among nurses to prevent musculoskeletal disorders. Numerous studies have identified relevant factors similar to the results of our study: female gender [41, 48], absenteeism [9], and psychosocial factors [8, 12, 16, 31, 32, 34, 35, 39, 42, 48–51]. In Vietnam, a previous study in Haiphong in Vietiep hospital (which have the largest number of nurses) also illustrated some factors as the gender, stress, and high age associated with MSDs [19], which was similar to our study outcomes.

This study has several limitations. Similar to many other studies using the Nordic Questionnaire, the process of data collection through interviews of events in the past 12 months may cause recall bias when answering the question, especially the recall of MSDs symptoms. Nevertheless, the investigators have been well trained and have tried to exploit these events carefully to limit this bias. In Vietnam, nursing work characteristics are divided into two major categories: clinical nursing, direct contact with the patient and related operations involving infusion or transport of patients; administrative nursing, nurses who do not contact patients and their work being only related to administrative procedures and patient records management. Our study was conducted on both types of nurses mentioned above with different work characteristics, so the prevalence of MSDs gained has not reflected all the characteristics of these different work types. However, research has still achieved this priority goal, which is to indicate the common prevalence of MSDs. In the exclusion criteria, our study was able to exclude other persistent musculoskeletal diseases from the beginning of the survey, this allowed us to assess current symptoms of MSDs without taking into account preexistent pathologies such as congenital spine disorders, trauma, and pain due to surgery or other diseases. Regarding logistic regression analysis, we were unable to cover all factors in the literature that could affect the MSDs such as working seniority, break time during working, division by particularly for each department, especially those with high working intensity, and unsuitable working positions (surgery, anesthesia, obstetric, and emergency). Nevertheless, the factors that we have exploited had been considering of their suitability in the context of our country and we hope to delve some wider range of factors in future studies.

The strong point of this study is that it is one of the first studies in Vietnam on work-related MSDs generally and on work-related MSDs among nurses specially. The scale of this study is quite large with high participation rates. The research population is highly representative for nurses in general, covering almost all nurses currently working in Haiphong city. This allows objective evaluation of the study results and reflects the current situation of MSDs among nurses in Vietnam. From there, it will make the premise and open up the prospects for further studies in Vietnam on professional MSDs specially and on occupational health generally.

## 5. Conclusion

A high prevalence of MSDs (74.7%) in nurses was found in this study, with the two most common sites being the lower back (44.4%) and the neck (44.1%). Some related factors were included: female gender, history of musculoskeletal diseases, absenteeism, and anxiety. More research will be needed in the future with more accurate data to provide the basis for future prevention measures to reduce the prevalence, incidence, and consequences of MSDs.

## Figures and Tables

**Table 1:** Sociodemographic characteristics of the study group, Haiphong, 2017.

Variables	N = 1179	n (%)	Mean	Median	Min-max	SD
Age (years)	< 45	1087 (92.2)	32.6	31.0	19 - 60	7.7
	≥ 45	92 (7.8)				

Gender	male	221 (18.7)				
	female	958 (81.3)				

BMI	< 18.5 underweight	134 (11.4)				
	18.5 – 22.9 normal	842 (71.4)				
	23 – 24.9 overweight	142 (12.0)				
	≥ 25 obesity	61 (5.2)				

Average income	< 3 million VND	281 (23.8)				
	3 – 5 million VND	679 (57.6)				
	5 – 7 million VND	180 (15.3)				
	7 – 10 million VND	37 (3.1)				
	≥ 10 million VND	2 (0.2)				

Marital status	single	192 (16.3)				
	married	972 (82.4)				
	divorced	8 (0.7)				
	separated	2 (0.2)				
	widowed	5 (0.4)				

Has children	yes	932 (79.1)				
	no	247 (20.9)				

Family member			4.2	4.0	1 - 20	1.3

Handed	left handed	72 (6.1)				
	right handed	1062 (90.1)				
	both hands	45 (3.8)				

History of MS disease	yes	132 (11.2)				
	no	1047 (88.8)				

VND: Vietnam dong; MS: musculoskeletal.

**Table 2:** Work characteristics and psychological distress level among nurses in Haiphong, 2017.

Variables	N	%	Mean	Median	SD	Min-max
Department						

Medicine	183	15.5				
Surgery	105	8.9				
Obstetrics & Gynaecology	91	7.7				
Pediatric	53	4.5				
Others	747	63.4				

Working position						

Administrative	111	9.4				
Clinical	1068	90.6				

Regularly work > 10 hours / day	438	37.2				

Working day / week	1179		5.3	5.0	0.5	5 - 7

Distance of duty (day)	805	68.3	5.2	5.0	1.5	1 - 8

Working hours / week	1179		49.5	48.0	11.3	20 - 99

Score on anxiety level	1179		4.5	4.0	3.7	0 – 19

**Table 3:** Absenteeism pattern during the last 12 months among nurses in Haiphong, 2017.

N= 1179		n (%)	Mean	Median	Min-max	SD
Absenteeism		573 (48.6)				

Number of absence			3.1	2	0 - 30	3.7

Total of absence (day)			24.7	5	1 -210	52.8

Cause of absence	long-term illness (over 2 weeks)	5 (0.9)				
	short-term illness (less than 2 weeks)	49 (8.6)				
	occupational disease	3 (0.5)				
	occupational accident	0 (0)				
	traffic accident	11 (1.9)				
	others (maternity leave, house work…)	516 (90.1)				

Working conditions lead to absent		37 (6.5)				

Desire to change positions or change jobs	never	884 (75)				
	rarely	114 (9.7)				
	sometimes	165 (14)				
	regularly	10 (0.9)				
	always	6 (0.5)				

**Table 4:** Factors associated with musculoskeletal disorders among nurses of district hospitals in Haiphong, 2017.

Independent variables		MSDs	Crude OR^a^	Adjusted OR^b^	*p*-value^c^
		n (%)	OR [IC 95%]	OR [IC 95%]*∗*	
Age	< 45	809 (74.4)	ref	ref	0.425
	≥ 45	72 (78.3)	1.2 [0.7 – 2.1]	1.3 [0.7 – 2.2]	

Gender	male	136 (61.5)	ref	ref	**<0.001**
	female	745 (77.8)	2.2 [1.6 – 3.0]	2.1 [1.5 – 2.9]	

Overweight	no	736 (75.4)	ref	ref	0.734
	yes	145 (71.4)	0.8 [0.6 – 1.1]	0.9 [0.7 – 1.4]	

Working hours/week	<40 hours	6 (60)	ref	ref	0.417
	≥40 hours	875 (74.9)	2.0 [0.6 – 7.1]	1.7 [0.5 – 6.5]	

History of musculoskeletal disease	no	756 (72.2)	ref	ref	**<0.001**
	yes	125 (94.7)	6.9 [3.2 – 14.9]	7.1 [3.2 – 15.5]	

Work position	administrative	85 (76.6)	ref	ref	0.669
	clinical	796 (74.5)	0.9 [0.6 – 1.4]	1.1 [0.7 – 1.9]	

Working > 10 hours/day	no regularly	548 (74)	ref	ref	0.168
	regularly	333 (76)	1.1 [0.8 – 1.5]	1.2 [0.9 – 1.7]	

Working day/week	≤5 days	664 (74.9)	ref	ref	0.691
	>5 days	217 (74.1)	0.96 [0.7 – 1.3]	0.9 [0.7 – 1.3]	

On duty	no	292 (78.1)	ref	ref	0.093
	yes	589 (73.2)	0.8 [0.6 – 1.02]	0.7 [0.5 – 1.1]	

Anxiety	no	538 (71)	ref	ref	**<0.001**
	yes	343 (81.5)	1.8 [1.3 – 2.4]	1.8 [1.3 – 2.4]	

Absenteeism	no	420 (69.3)	ref	ref	**<0.001**
	yes	461 (80.5)	1.8 [1.4 – 2.4]	1.7 [1.3 – 2.3]	

^a^Univariate analysis; ^b^multiple logistic regression; ^c^likelihood-ratio test.

MSDs: musculoskeletal disorders; OR: odds ratio; CI: confidence interval.

*α* < 0.05.

## Data Availability

The EXCEL/SPSS data used to support the findings of this study are available from the corresponding author upon request.
